# Productivity gains in vegetables from rice husk biochar application in nutrient-poor soils in Timor-Leste

**DOI:** 10.1038/s41598-023-38072-2

**Published:** 2023-07-05

**Authors:** Rob Williams, Joao Bosco Belo, Julieta Lidia, Salvador Soares, Decio Ribeiro, Celestino L. Moreira, Luis Almeida, Louise Barton, William Erskine

**Affiliations:** 1 Institute of Agriculture/School of Agriculture and Environment, University of Western Australia, 35 Stirling Hwy, Nedlands, WA 6009 Australia; 2 AI-Com Project, PO Box 221, Dili, Timor-Leste; 3 Ministry of Agriculture, Forestry and Fisheries, Avenida Presidente Nicolau Lobato, Comoro, Dili, Timor-Leste

**Keywords:** Ecology, Plant sciences

## Abstract

Response to fertilisation with biochar is greatest in field crops on acidic tropical soils, but limited information is available for vegetable crops. As a case-study using vegetable production in Timor-Leste, we assessed if biochar alleviates nutritional constraints to vegetables in low-nutrient soils. Field trials on vegetable crops were conducted with fertiliser combinations of rice husk biochar, phosphate and local fertiliser at three sites. A pot soil incubation trial of biochar was undertaken with soil from the acid site, where rice husk biochar had a larger effect on productivity than the other fertilisers in chili pepper, tomato and soybean with an average yield increase with biochar of 230% over control. Combining phosphate with biochar augmented the yield over biochar alone in chili pepper, tomato and soybean. At neutral and alkaline sites, fertilisation with biochar lifted mean yield over the control. Soil constraints alleviated by fertiliser were primarily from P and Zn deficiencies. Marked increases in vegetable yields, among the highest globally, were achieved with fertilisation with biochar individually and in combination with phosphate in low nutrient soil in Timor-Leste. Clearly, rice husk biochar is a promising avenue to fertilise the soil with P and Zn and increase crop productivity in Timor-Leste.

## Introduction

Biochar is a carbon-rich product from the thermo-chemical decomposition of biomass under limited oxygen conditions. Its use to amend soil has been reported to have multiple benefits in an extensive literature, and especially in the improvement of crop production in highly degraded and nutrient-poor soils^1–4^. A global-scale meta-analysis showed that biochar has, on average, no effect on crop yield in temperate latitudes, but elicits a 25% average increase in yield in the tropics where acid soils are widespread^4^. Acidic soils were more responsive to biochar application than alkaline soils, and the effect systematically declined as initial pH increased^4^. Biochar soil fertilisation increases crop productivity through the improvement of soil physical properties such as permeability, water holding capacity and nutrient use efficiency^3^, and through its liming effect on acid soils^5^. In summary, research shows that productivity gains from soil fertilisation with biochar have been greatest in tropical acidic low-nutrient soils^2–4^.

Adoption of biochar in agriculture is expected to be greatest for high market value commodities, such as horticultural crops, that have shown substantial positive responses to biochar additions under some circumstances^2^. To-date, most of the research on soil fertilisation with biochar has been conducted on cereal and tuber crops and there are few studies of the potential of soil biochar fertilisation in horticultural systems where the yield responses have been variable. Under a temperate climate in Tasmania in a high-input system, applying blue mallee (*Eucalyptus polybractea* L.) biochar to a fertile soil improved neither soil quality nor cauliflower, pea and broccoli crop productivity^6^. In Northeast China peanut shell biochar alone or in co-application with N fertiliser had little effect on vegetable (rape (*Brassica napus* L.), lettuce (*Lactuca sativa* var. *longifolia* L.) and pakchoi (*Brassica chinensis* L.) yield in four consecutive seasons^7^. In a Mediterranean climate in Iran, biochar fertilisation improved soil structure, water use efficiency and pumpkin yield^8^. In tropical Paua New Guinea biochar (rice husk) fertilisation improved the total tuber yield of sweet potato by approximately 20%, while co-application with mineral fertilisers augmented total tuber yield by 100%^9^. In Ghana by adding biochar (rice husks and corn cobs) to their normal agricultural practice farmers were able to increase lettuce yields by 93%^10^. In summary, in cool climates response to biochar has been variable in horticultural crops, whereas in warmer tropical climates biochar responses have been substantial.

Timor-Leste is a small tropical country with a population of approximately 1.3 million, predominantly (85%) engaged in agriculture. The national diet is dominated by the cereals maize and rice and by the tubers of sweet potato and cassava^11^. Soils are sedimentary with high clay contents and pH is generally alkaline, although some soils formed in older geological formations are slightly acidic^12^. In Timor-Leste soil constraints restricting yields variously include high (> 8) and low pH (< 5.5), and inadequate plant available N and P. For example, maize yields tend to be greater in soils with intermediate pH values (5.5–8) than low (< 5.5) or high (> 8) values^13^. Acid soils are concentrated at higher elevations with mean soil pH decreasing with elevation at 1.5–2.0 pH units per 1000 m^−1^ elevation^13^. A 2013 survey of 195 farm soils in Timor-Leste showed low concentrations of Olsen P (< 10) are a problem in 73% of Timor-Leste cropping soils^14^. Low concentrations of plant available nitrogen are also considered to restrict crop yields nationally^15^.

The need for fertilisers and liming to increase yields to feed the growing population is becoming increasingly pressing for national food security in Timor-Leste. Typically smallholder farmers in Timor-Leste apply little fertiliser or organic matter to their crops^16^ with farmyard and chicken manure the most common fertiliser source. Chemical fertilisers are not available to most subsistence farmers and hence not widely used in most traditional cropping systems^17^. Instead, chemical fertiliser use is limited to some rice and vegetable farms. The liming of acid soils is unknown in Timor-Leste as lime is unavailable for sale, and so there is no method currently used to decrease the acidity of low pH soils. An alternative, and economically viable, approach is needed to improve the chemical fertility of Timor-Leste’s cropping soils. Recently, large and significant responses to rice husk biochar and phosphate (SP36) were recorded on the North Coast at Vemasse, growing crops after rice in a high pH low P soil^18^. We deduced that similar positive responses to added biochar would be replicable in upland soils within Timor-Leste, based on the review of Jeffrey et al.^4^ and a knowledge of rice husk biochar chemistry of Prakongkep et al.^19^, who suggested that this biochar could have value as a multi-nutrient fertiliser and by neutralising acid soils.

We hypothesized that the use of biochar fertilisation in nutrient-poor tropical soils would provide ideal preconditions both to increase horticultural productivity across a wide range of pH and to decrease acidity in acid soils. Accordingly, as a case study using a range of upland low-nutrient soils in Timor-Leste, this research asked if vegetable productivity can be improved through soil fertilisation using locally-produced rice husk biochar within field-based experiments. Then, using soil from the most acid and responsive site in an incubation pot experiment, can we understand the prevailing soil constraints as well as the response to applied biochar.

## Results

### Field study

**Figure 1:**
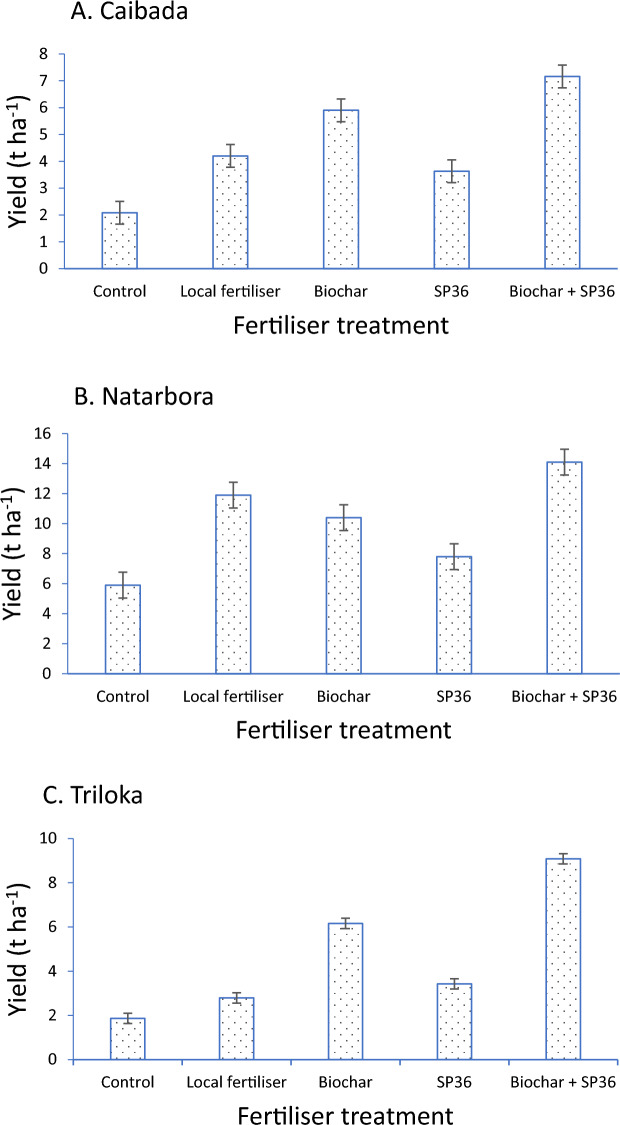
Mean yield (t ha^−1^) over crops of response to fertiliser treatments (1. Control (no fertiliser application); 2. Local fertiliser regime (see details below); 3. Fertilisation with rice husk biochar (20 t ha^−1^ at Triloka and 30 t ha^−1^ at Caibada and Natarbora); 4. Addition of SP36 (28.8 kg P ha^−1^ and 4 kg S ha^−1^); and 5. The combination of biochar (Treatment 3) and SP36 (Treatment 4) at (**A**) Caibada, (**B**) Natarbora and (**C**) Triloka study locations. The local fertiliser treatment varied by site according to local usage: At Caibada 10 t cow manure ha^−1^ applied; at Natarbora 100 kg NPK (15:15:15) with 2 t cow manure ha^−1^; and at Triloka 1.2 t cow manure ha^−1^. Standard errors are indicated as error bars.

Crop growth and yield: At Caibada—the alkaline site, (Table 1, Fig. 1A) control plot yields were 1.8 t ha^−1^ in chili pepper, 1.5 t ha^−1^ in sweet pepper, 4.1 t ha^−1^ in Phaseolus beans, and 0.9 t ha^−1^ for carrots. The effect of fertiliser treatment on yield was significant and consistent over the vegetable crops (*P* < 0.05), as the fertiliser x crop interaction was non-significant. The addition of 10 t cow manure or SP36 ha^−1^ only increased productivity (*P* < 0.05) in carrot from the control and responses in the other crops did not reach significance. Biochar application and the combination treatment of biochar with SP36 increased yield relative to the control in all crops (*P* < 0.05), with an overall average increase of 183% from biochar alone. This comprised an increase in chili pepper to 5.1 t ha^−1^ with biochar, in sweet pepper to 5.0 t ha^−1^, in Phaseolus bean to 9.9 t ha^−1^ and in carrot to 3.7 t ha^−1^. The productivity increases in the peppers were primarily through increased numbers of fruits per plant, whereas in carrots the biochar induced yield increase came from a tripling of average taproot weight from 36.5 to 112 g (Table 1). At the alkaline site there was no advantage of the combination treatment of biochar in combination with SP36 over biochar alone.

**Table 1:** Crop yield, yield components, plant height and the number of fully open leaves at weeks 2 and 4 of transplanting of four vegetable crops (chili pepper, sweet pepper, Phaseolus bean and carrot) in response to fertiliser treatments (control, local fertiliser, biochar, SP36, and biochar + SP36 in combination) at Caibada in 2018.

		Treatment							
Crop	Character	Control	*Local fertiliser	Biochar	SP36	Biochar + SP36	*P* value	SE	CV%
Chili pepper	Yield (t ha^−1^)	1.75 a	3.27 ab	5.07 bc	2.3 a	6.07 c	0.018	0.75	35.4
	Density (pl m^−2^)	3.3	3.2	3.2	3.3	3.3	> 0.05		3.4
	No. fruits pl^−1^	74.8 a	124.7 b	173.6 c	123.8 b	261.1 d	< 0.001	11.38	13.0
	Fruit weight (g)	1.12	1.35	1.26	1.21	1.25	> 0.05		24.1
	*Height at 2 wk (cm)	6.4	7.1	10.1	8.5	13.3	> 0.05		30.2
	*Height at 4 wk (cm)	10.3	14.6	17	11.7	22.4	> 0.05		29.8
	*No. leaves at 2 wk pl^−1^	7.4 a	8.4 ab	10.6 c	9.3 bc	12.4 d	0.002	0.551	9.9
	*No. leaves at 4 wk pl^−1^	10.3	15.2	16.7	12.3	18.7	> 0.05		24
Sweet pepper	Yield (t ha^−1^)	1.54 a	3.21 a	4.97 b	2.9 a	5.68 b	0.004	0.54	25.4
	Density (pl m^−2^)	3	3.07	3.15	3.15	3	> 0.05		3.7
	No. fruits pl^−1^	1.7 a	3.7 b	5 c	3 b	5.7 c	< 0.001	0.36	16.2
	Fruit weight (g)	43.5	50.7	52.0	57.8	60.5	> 0.05		20.5
	*Height at 2 wk (cm)	10.7 a	15 bc	16.2 bc	12.9 ab	17.7 c	0.01	1.05	12.5
	*Height at 4 wk (cm)	14.2 a	17.9 b	20.4 b	18.7 b	23.5 c	< 0.001	0.897	8.2
	*No. leaves at 2 wk pl^−1^	9	11.3	12.8	11.6	13.3	> 0.05		12.8
	*No. leaves at 4 wk pl^−1^	16.7	15.6	21.3	17.1	22.4	> 0.05		16.4
Phaseolus bean	Yield (t ha^−1^)	4.12 a	7.02 ab	9.85 bc	7.12 a	12.20 c	0.019	1.298	27.9
	Density (pl m^−2^)	3.2	3.3	3.3	3.3	3.3	> 0.05		2.5
	No. pods pl^−1^	27.2 a	38.1 ab	54.1 bc	41.2 ab	67.9 c	0.006	5.49	20.8
	Fresh pod weight (g)	4.93	4.87	5.6	5.46	5.45	> 0.05		20.0
	Height at 2 wk (cm)	9.6 a	13.2 bc	15.4 cd	12.1 b	16.3 d	< 0.001	0.715	9.3
	Height at 4 wk (cm)	97 a	119 b	138 b	134 b	158 c	< 0.001	0.061	8.0
	No. leaves at 2 wk pl^−1^	4 a	4.2 b	4.6 c	4.3 b	5 d	< 0.001	0.061	2.4
	No. leaves at 4 wk pl^−1^	13 a	14 a	17.7 b	15.6 ab	20.6 c	< 0.001	0.792	8.5
Carrot	Yield (t ha^−1^)	0.92 a	3.31 bc	3.71 bc	2.1 ab	4.7 c	0.011	0.56	33.0
	Density (pl m^−2^)	34.3	37.9	35.8	35.7	35.4	> 0.05		7.1
	No. taproots pl^−1^	1	1	1	1	1	> 0.05		16.4
	Taproot weight (g)	36.5 a	83.7 abc	112.2 bc	60.5 ab	98.1 bc	0.012	15.6	30.7
	*Height at 2 wk (cm)	6.8 a	8.2 b	9.7 cd	9 bc	11 d	< 0.001	0.418	8.1
	*Height at 4 wk (cm)	11.4 a	13.5 b	15.2 bc	14.1 b	16.9 c	0.003	0.615	7.5
	*No. leaves at 2 wk pl^−1^	3 a	3.2 ab	3.4 bc	3.4 bc	3.8 bc	0.014	0.117	6.0
	*No. leaves at 4 wk pl^−1^	3.3 a	3.8 b	4.1 b	3.9 b	4.6 c	< 0.001	0.118	5.2

Values are means of three replicates, and treatments followed by the same letter and in the same row are **not** significantly different from each other (*P* < 0.05). Standard Error (SE) is given when the treatment effect was significant (*P* < 0.05). * 10 t cow manure ha^−1^; * Weeks after transplanting.

At Caibada, fertiliser effects were visible early and while plants were still seedlings. Two weeks after transplanting there were already significant increases from fertiliser in the number of expanded leaves in all crops except sweet pepper and in plant height for all crops bar chili pepper (Table 1).

At Natarbora, tomato production was 5.9 t ha^−1^ in the control plots (Table 2, Fig. 1B). The application of farmer rate of fertiliser (100 kg NPK (15:15:15) ha^−1^ + 5 t ha^−1^ cow manure) and of biochar alone approximately doubled tomato yield over the control. Equally high tomato productivity was achieved with the application of biochar combined with SP36 fertiliser. Tomato plant density, number of fruits per plant and fruit weight did not vary significantly with fertiliser treatment (*P* > 0.05; Table 2).

**Table 2:** Tomato yield and yield components in response to fertiliser treatments (control, local fertiliser, biochar, SP36 (P_2_0_5_), and biochar + SP36 in combination) at Natarbora in 2018.

	Treatment							
Character	Control	*Local fertiliser	Biochar	SP36	Biochar + SP36	*P* value	SE	CV %
Yield (t ha^−1^)	5.9 a	11.9 bc	10.4 bc	7.8 ab	14.1 c	0.013	0.86	22.2
Density (pl m^−2^)	2.7	2.7	2.7	2.7	2.7	> 0.05		22.2
No. fruits pl^−1^	62.7	92.1	95.9	79.5	115.2	> 0.05		33.2
Fruit weight (g)	34.2	45.3	38.1	37.2	45.2	> 0.05		19.7

Values are means of three replicates, and treatments followed by the same letter and in the same row are **not** significantly different from each other (*P* < 0.05). Standard Error (SE) is given when the treatment effect was significant (*P* < 0.05). * 100 kg NPK (15:15:15) with 2 t cow manure ha^−1^.

At Triloka—the acid site, control plot yields were 2.2 t ha^−1^ in chili pepper, 4.5 t ha^−1^ in tomato, and 0.4 t ha^−1^ in both soybean and yard-long bean. The effect of crops, fertiliser treatments and crop x fertiliser on yield were all significant (*P* < 0.001). Furthermore, the fertiliser effect mean square was significantly greater than the mean square of the interaction effect (*P* < 0.01) indicating the over-arching effect of fertiliser on productivity across crops. Local fertiliser application (2 t cow manure ha^−1^) increased yield significantly over the control for only tomato to 7.0 t ha^−1^ (*P* < 0.05) (Table 3, Fig. 1C). Rice husk biochar application increased—compared to the control—chili pepper yield to 6.8 t ha^−1^, tomato yield to 13.9 t ha^−1^, soybean yield to 1.1 t ha^−1^, and yard-long bean yield to 2.8 t ha^−1^. The application of SP36 fertiliser significantly increased tomato and soybean yield relative to the control (*P* < 0.05). Analysis of yield components showed that in all crops the biochar effect was delivered through a combination of increases in both the numbers of fruits/plant and the average fruit size (Table 3). Combining SP36 with biochar significantly augmented the yield over biochar alone in chili pepper, tomato and soybean.

**Table 3:** Crop yield, yield components, plant height and the number of fully open leaves at weeks 2 and 4 of transplanting of four vegetable crops (chili pepper, tomato, soybean and yard-long bean) in response to fertiliser treatments (control, local fertiliser, biochar, SP36, and biochar + SP36 in combination) at Triloka in 2018.

		Treatment							
Crop	Character	Control	*Local fertiliser	Biochar	SP36	Biochar + SP36	*P* value	SE	CV %
Chili pepper	Yield (t ha^−1^)	2.19 a	2.86 a	6.84 b	4.03 a	13.14 c	< 0.01	0.23	17.3
	Density (pl m^−2^)	1.8 a	2.3 a	3.3 b	2.7 a	3.3 b	< 0.01	0.25	15.8
	No. fruits (pl^−1^)	9.0 a	9.8 a	24.4 c	12.6 b	36.8 d	< 0.01	1.31	12.3
	Fruit weight (g)	5.9 a	7.1 a	9.5 b	7.2 a	9.8 b	< 0.01	0.58	12.8
	*Height at 2 wk* (cm)	6.0 a	6.6 a	8.6 b	6.6 ab	9.7 b	< 0.001	0.349	8.1
	*Height at 4 wk* (cm)	11.7 a	13.8 b	25.4 c	15.2 b	28.4 d	< 0.001	0.535	4.9
	*No. leaves at 2 wk*pl^−1^	4.7 a	4.9 a	6.9 b	6.6 b	8.1 c	0.001	0.23	6.4
	*No. leaves at 4 wk*pl^−1^	6.4 a	6.3 a	9.3 b	6.9 a	10.8 c	< 0.001	0.317	6.9
Tomato	Yield (t ha^−1^)	4.49 a	7.03 b	13.92 c	7.59 b	18.7 d	< 0.01	0.23	17.3
	Density (pl m^−2^)	2.07	2.11	2.22	2.22	2.22	> 0.05		15.8
	No. fruits pl^−1^	12.4 a	14.3 b	27.1 d	17.7 c	36.6 e	< 0.01	1.53	12.3
	Fruit weight (g)	26.0 a	32.8 b	58.2 d	38.4 c	67.7 e	< 0.01	3.3	12.8
	*Height at 2 wk (cm)	12.7 a	14.1 ab	19.1 cd	17.1 cd	23.8 d	0.002	1.33	13.1
	*Height at 4 wk (cm)	18.1 a	20.3 ab	27.4 c	23.1 b	31.1 c	< 0.001	1.193	8.6
	*No. leaves at 2 wk pl^−1^	5.6	5.8	7.1	7.1	7.9	> 0.05		13.6
	*No. leaves at 4 wk pl^−1^	7.7 a	7.8 a	9.4 b	8.1 a	12.9 c	< 0.001	0.238	4.5
Soybean	Yield (t ha^−1^)	0.39 a	0.44 a	1.1c	0.74 b	1.88 d	< 0.01	0.23	17.3
	Density (pl. m^−2^)	6.3 a	6.4 ab	7.0 bc	6.6 ab	7.2 c	< 0.01	0.61	15.8
	No. pods pl^−1^	7.1 a	9.7 b	22.9 d	12.4 c	29.6 e	< 0.01	1.16	12.3
	100 seed weight (g)	16.0 a	18.0 b	21.3 d	19.7 c	22.6 e	< 0.01	0.43	3.8
	Height at 2 wk (cm)	10.6 a	10.6 a	12.9 b	10.3 a	15.1 c	< 0.001	0.206	3.0
	Height at 4 wk (cm)	16.0 a	17.3 a	25.1 bc	21.3 b	27.6 c	< 0.001	1.19	9.6
	No. leaves at 2 wk pl^−1^	4.7 a	5.1 ab	5.5 b	5.4 b	7.0 c	< 0.001	0.214	6.7
	No. leaves at 4 wk pl^−1^	7.8 a	8.7 a	11.9 b	8.7 a	12.3 b	0.026	0.934	16.4
Yard-long bean	Yield (t ha^−1^)	0.39 a	0.83 ab	2.78 b	1.34 ab	2.6 1b	< 0.01	0.23	17.3
	Density (pl m^−2^)	4.9 a	4.4 a	6.0 b	5.3 a	7.6 c	< 0.01	0.52	15.8
	No. pods pl^−1^	1.7 a	2.2 a	5.6 b	2.1 a	5.3 b	< 0.01	0.24	12.3
	Fruit weight (g)	4.5 a	5.6 a	15.2 c	10.4 b	12.0 b	< 0.01	0.71	12.8
	Height at 2 wk (cm)	8.4 a	8.2 a	17.2 b	8.8 a	14.4 b	0.013	1.648	25.0
	Height at 4 wk (cm)	14.3 a	14.4 a	33.0 b	17.2 a	29.4 b	< 0.01	2.078	16.6
	No. leaves at 2 wk pl^−1^	2.7 a	2.7 a	3.4 b	2.8 a	3.6 b	0.035	0.21	12.0
	No. leaves at 4 wk pl^−1^	4.6 a	4.7 a	6.9 c	4.8 a	5.8 b	< 0.001	0.209	6.8

Values are means of three replicates, and treatments followed by the same letter and in the same row are **not** significantly different from each other (*P* < 0.05). Standard Error (SE) is given when the treatment effect was significant (*P* < 0.05). *2 t cow manure ha^−1^; *Weeks after transplanting.

At Triloka—as at the alkaline site Caibada—applying biochar fertiliser positively increased plant height in all crops as early as 2 weeks after transplanting and the number of open leaves in chili pepper, soybean and yard-long bean (Table 3).

Soil analyses of field samples: Means of soil pH and Mehlich 3 soil extraction analyses from bulk plot soil samples after harvest of fertiliser treatments at three sites (Caibada, Natarbora and Triloka) are given in Supplementary Table 2. The interaction of site x fertiliser treatment was non-significant (*P* > 0.05) for pH and most elements (Al, Ca, Cu, K, Mg, Na, S and Zn) except for Fe and P. For these two elements, the interaction was primarily caused by positive responses to the combined fertiliser treatment (biochar plus SP36) at the Natarbora site. Surprisingly, despite the large agronomic responses to applied fertilizer there were no significant effects of the fertiliser treatments on pH and most elements (Al, Ca, Cu, K, Mg, Na, S and Zn) recorded in the bulk soil samples. There were highly significant differences between the sites for all soil parameters tested (Supplementary Table 2).

### Soil incubation pot study

**Figure 2:**
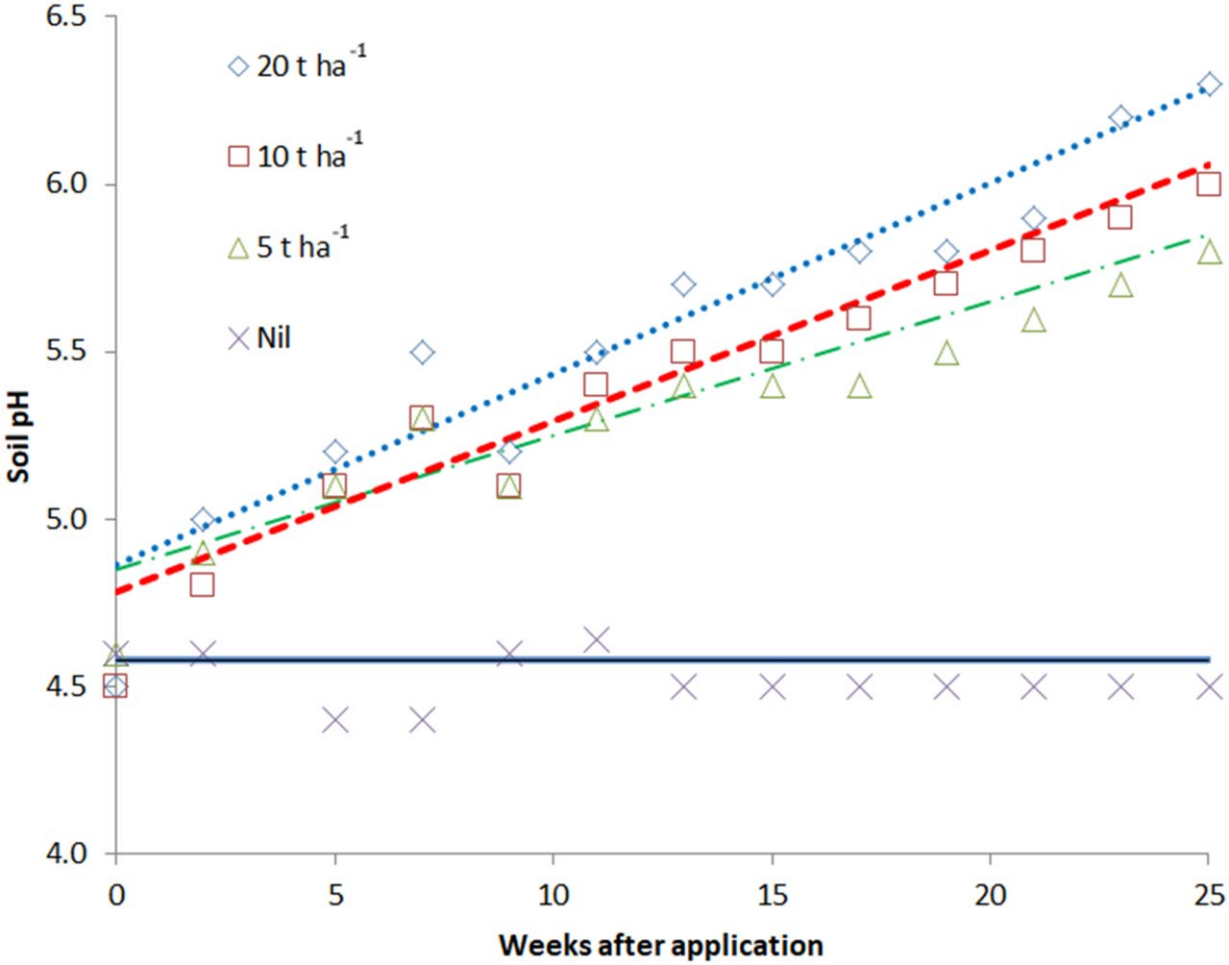
Response in soil pH to different biochar application rates (0, 5, 10 and 20 t ha^−1^) for 26 weeks after application in the soil incubation trial. Interaction LSD (*P* < 0.05) = 0.18.

In a soil incubation pot study—without plants—of fertilisation with rice husk biochar**, a**pplying biochar increased soil pH linearly over time for all application rates, but more as we increased the application rate (Fig. 2). For example, starting from an initial soil pH of 4.5, after 25 weeks of incubation the pH with 5 t biochar ha^−1^ was 5.6, 10 t ha^−1^ was 5.9, and 20 t ha^−1^ was 6.2. Fitting linear regressions to soil pH values over time for each concentration of added biochar accounted for 83% of the variation in soil pH.

Biochar with its high carbon content (7) increased % soil C and had lesser impact on nitrogen content by weeks 21 and 23 of incubation (Table 4). Olsen P concentrations increased with increasing biochar fertilization after 21- and 23-weeks incubation. The extract of rice husk biochar using a Mehlich 3 extract showed a P concentration of 561 mg kg^−1^ (Table 3). The application of this biochar significantly increased soil available P (Mehlich 3) of y = 0.2429x + 8.5 (R^2^ = 0.98) from the final two dates samples of the incubation (Table 4). This is consistent when considering the changes in Olsen P concentration, as applying biochar more than doubled Olsen P from 1.2 (control) to 3.3 ppm at 20 t ha^−1^.

**Table 4:** Mean of samples (Samples) at weeks 23 and 25 from soil analyses (pH, C and N content, and Mehlich 3 extractions) of the effect of rice husk biochar application rates (Rate: 0, 5, 10 and 20 t ha^−1^) in an incubation pot trial on an acid soil from Triloka.

Variable	Rates of biochar (t ha^−1^)				fProb			CV %	LSD (5%) Biochar
	0	5	10	20	Rate	Samples	Rate x samples		
pH*	4.6 a	5.8 b	6.0 c	6.3 d	< 0.001	0.025	0.493	1.8	0.09
%N	0.213 a	0.226 a	0.226 a	0.239 b	0.003	0.122	0.97	5.6	0.0093
%C	3.1 a	3.7 ab	4.3 b	5.5 c	< 0.001	0.915	0.615	25.4	0.83
P Olsen*	1.2 a	1.6 b	2.0 c	3.3 d	< 0.001	0.479	0.999	15.6	0.28
Al mg kg^−1^	1280 b	1293 b	1205 a	1165 a	0.011	0.483	0.227	7.4	80.5
Ca mg kg^−1^	2391	2530	2548	2543	0.154	0.646	0.299	7.1	ns
Cu mg kg^−1^	2.83	2.91	2.85	2.84	0.623	0.82	0.78	7.0	ns
Fe mg kg^−1^	59.1	60.3	61.1	63.5	0.251	0.531	0.534	9.0	ns
K mg kg^−1^	104 a	150 ab	207 b	297 c	< 0.001	0.687	0.248	49.5	77.1
Mg mg kg^−1^	407	411	425	431	0.477	0.11	0.168	7.8	ns
Na mg kg^−1^	40.1	41.3	45.9	41.4	0.366	0.185	0.086	20.1	ns
P mg kg^−1^	8.7 a	9.4 ab	11.0 b	13.4 c	0.001	0.513	0.278	25.2	2.1
S mg kg^−1^	40.2	38.5	35.5	34.9	0.324	0.006	0.025	15.3	ns
Zn mg kg^−1^	1.8 a	3.0 ab	4.9 b	7.9 c	0.001	0.68	0.416	80.2	2.42

*Dili soil laboratory.

Values are means of five replicates, and treatments followed by the same letter and in the same row are **not** significantly different from each other (*P* < 0.05). Units in Table are mg kg^−1^ for Al, Ca, Cu, Fe, K, Mg, Na, P, S, and Zn. Additional analyses are for nitrogen (N), carbon (C) as %, and P Olsen in ppm.

Applying biochar increased extractable K and Zn concentrations in the soil after incubation. The extract of rice husk biochar contained high concentrations of both K at 4144 mg kg^−1^ and Zn at 7.0 mg kg^−1^ (Table 3). Adding biochar increased soil K availability significantly from 104 mg K kg^−1^ in the control to 297 mg kg^−1^ with 20 t ha^−1^ biochar. Similarly, rice husk biochar application increased Zn from 1.8 mg Zn kg^−1^ in the control to 7.9 mg kg^−1^ with the application of 20 t ha^−1^ biochar, with Zn increasing by 1.9 mg kg^−1^ t^−1^ biochar applied (R^2^ = 0.99).

Biochar application had no significant effect on soil Ca, Cu, Fe, Mg, Na and S concentrations. There was a small, but significant reduction in soil available Al with biochar application (Table 4).

**Figure 3:**
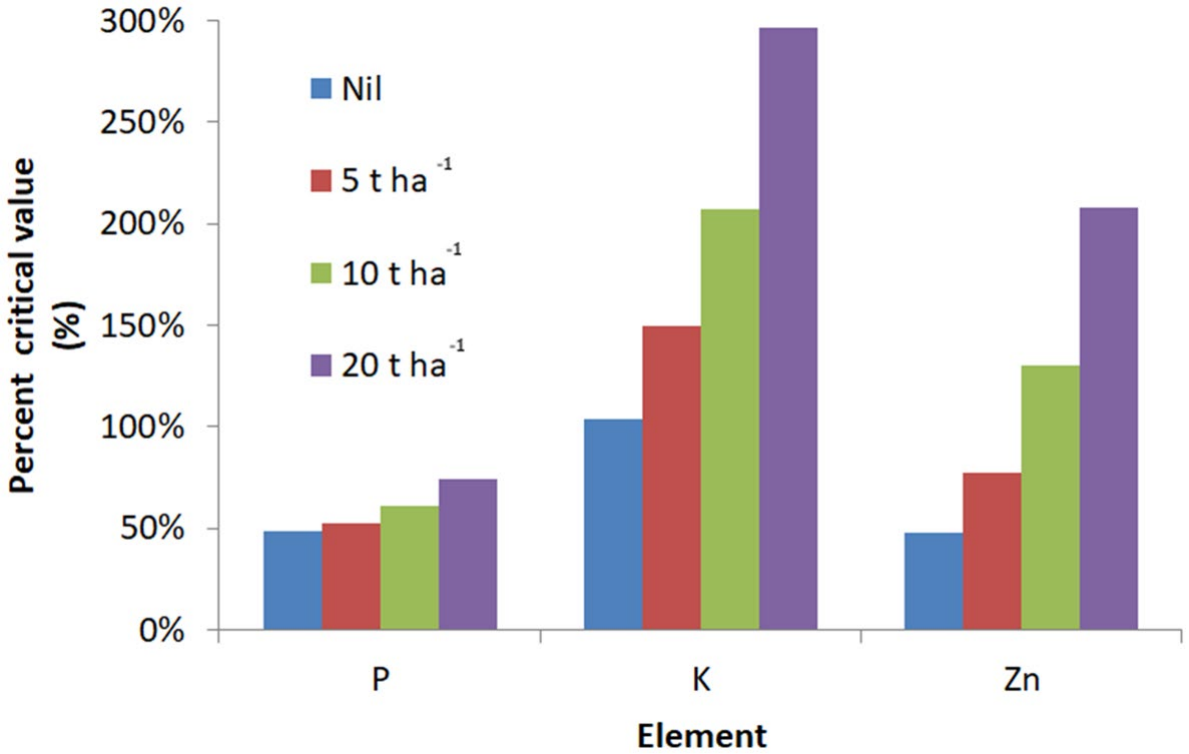
Changes in available P, K and Zn in an acid soil incubated for 26 weeks with four rates of biochar application (0, 5, 10 and 20 t ha^−1^). Data shown are as a percentage of the critical concentrations of 18, 100 and 3.8 mg kg^−1^ for Mehlich 3 extractable P, K and Zn, respectively, based on Wortmann et al. (2019).

The increase of extractable concentrations of P, Zn and K in response to biochar application were compared to soil critical values published by Wortman et al.^20^ in Fig. 3. The soil extractable concentrations of P remain below the critical soil concentration of extractable P (Mehlich 3) (18 mg kg^−1^), even with the application of 20 t ha^−1^. The addition of rice husk biochar increased extractable soil Zn concentrations from 48% of the critical concentration (3.8 mg kg^−1^) in the control pots to more than double the critical value with the maximum application of 20 t ha^−1^ rice husk biochar. In the case of K, the control soil had an extractable (104 mg kg^−1^) just above the critical soil concentration (100 mg kg^−1^). The application of rice husk biochar increased the soil extractable concentration to 297% of the critical value.

## Discussion

The yield responses from fertilisation with rice husk biochar in this study are among the highest recorded in the literature for horticultural crops^2–4^. The response to biochar application was seen not only in acid soil but also in neutral and alkaline soils. The biochar response was primarily due to a fertilisation effect as a source of nutrients. Our findings are consistent with Prakongkep et al.^19^ and demonstrated the value of rice husk biochar as a multi-nutrient fertiliser. Jeffrey et al.^4^ in a global-scale meta-analysis showed that biochar increases in yield in the tropics are by fertilisation and a liming effect.

A major horticultural response to biochar was observed not only in the acid soil—as anticipated from the literature^4,21^—but also in neutral and alkaline tropical soils. Thus, at the acid site (Triloka) the biochar treatment yielded 212% more than the control in chili pepper, 210% in tomato, 182% in soybean and 612% in yard-long bean. Whilst, at the neutral site (Natarbora) the biochar treatment yielded 76% more tomato yield than the control; and at the alkaline soil site (Caibada) the biochar treatment yielded 189% more than the control in chili pepper, 223% in sweet pepper, 139% in Phaseolus bean and 303% in carrot.

In this study the major agronomic responses to fertilisation with rice husk biochar stem primarily from its action as a source of nutrients and modifying soil pH. A review by Ding et al.^22^ indicated four ways that biochar can increase soil fertility: (1) biochar as a source of nutrients; (2) adsorption and desorption of nutrients on biochar; (3) the influence of biochar on soil properties; and (4) the effects of biochar on soil biota. Support for biochar as a source of nutrients in this study comes from two strands of evidence. The first is that the observable impact of biochar application on crop growth and final yield was visible very soon after planting/transplanting. At just 2 weeks after planting/ transplanting, seven of the eight crops across the two sites (Caibada and Triloka) were significantly taller and six of the eight crops had more leaves than the control with biochar fertilisation. Clearly, the mechanism behind the large yield response is fast acting. The second line of evidence is that the concentrations of extractable ions in the rice husk biochar correlated with changes in the extractable concentrations in the soil incubation experiment of the acid soil site. Additionally, just in the acid soil site, a secondary reason for the yield response was the liming effect from the addition of the alkaline rice husk biochar (pH 8.9), which lifted soil pH in the soil incubation trial. As the dosage of biochar increased, soil pH increased at a higher rate over time. Considering final pH levels, the response to applied biochar at 20 t ha^−1^ was well described (R^2^ = 0.95) by a quadratic equation to increase from pH 5.2 to reach a maximum of pH = 6.3. Notwithstanding this liming effect, as the biochar effect was observed on neutral and alkali soils—as well as acid soil, the fertilisation effect of biochar is of paramount importance and its liming effect just implicated in the acid soil.

At the acid site, yields from the combination of biochar with SP36 further enhanced crop growth and yield of specific crops beyond the biochar alone response. Yields of this combination treatment were 92% more than the sole biochar treatment in chili pepper, 34% in tomato, and 71% in soybean. At the other two sites the yield advantage of the combination treatment did not reach the level of significance at *P* = 0.05. Comparing the yields of the combination treatment (biochar + SP36) against the yield of sole SP36 application, the combination treatment out-yielded that from SP36 application alone by 226% in chili pepper, by 146% in tomato, and 154% in soybean. Rice husk biochar specifically can improve soil fertility and improve the effectiveness of fertilizer when mixed due to its nutrient retention capacity and high silica content^23^. Meta-analysis found that, on average, biochars increase P availability by a factor of 4.6^24^. Specifically, at our acid site there was a deficiency not only in P but also in other nutrients.

To identify which nutrients were deficient, two approaches were followed: firstly through a comparison of the control plot soils at the three sites against typical tropical soil critical values for different elements and secondly by the incubation trial of the acid Triloka soil. As there is no history of analysing and interpreting Mehlich 3 extracts in Timor-Leste, critical values were sourced from a review of croplands of tropical Africa^20^. The review suggested critical soil test values were 18, 100, 1000, 100, 8, 3.8, and 1.2 mg kg^−1^ for P, K, Ca, Mg, S, Zn and Cu, respectively. These values were used as benchmarks for Mehlich 3 extracts in this study of tropical soils in Timor-Leste. Comparison of these critical values with our three experimental soils showed the test soils as adequate in the elements Ca, Cu, Mg and S, but lacking at all three sites in Zn and at some sites for P and K. In the soil incubation trial, the extent that biochar increased the concentrations of extractable nutrients relative to the critical concentration, differed between nutrients (Fig. 3). The extractable concentrations of the nutrients (P, K and Zn) increased in a linear fashion for all three nutrients in the soil incubation trial. The application of the rice husk biochar gave a highly significant response in soil available P (Mehlich 3) of y = 0.2429x + 8.5 (R^2^ = 0.98) and zinc of (y = 1.66* x + 0.31, R^2^ = 0.995) from the final two samples. Considering Olsen P, biochar fertilisation more than doubled P from 1.2 ppm (control) to 3.3 at 20 t ha^−1^. For both P and Zn, the extractable concentrations in the control soil were also well below the critical concentrations suggested by Wortmann et al.^20^. The increase in soil concentrations of both elements was insufficient to meet the critical soil concentration for P with 20 t ha^−1^ added, but soil Zn concentrations easily exceeded the critical concentration for Zn with an application rate of 10 t ha^−1^ of rice husk biochar. Extractable concentration of K in the control soil were above the critical value, and K soil concentrations increased to more than 300% of the critical concentration with the addition of 20 tha^−1^. Collectively, this suggests that in the acid Triloka soil the response to biochar and P is also due to P and Zn. The concentrations of K seem adequate based on a critical value of 100 mg K kg^−1^.

Among the few analyses of soil constraint analyses previously reported for Timor-Leste, extremely low concentrations of P and Zn were noted for cassava at some sites by Howeler^25,26^. Across the 48 agricultural soils listed by Howeler^25,26^ in Timor Leste, a significant percent was deficient in P (38%), Zn (58%) and one or both Zn and P (74%). The soil P test used was P in Bray II, with a critical limit of 5 µg g^−1^. A subsequent survey of 195 agricultural soils in Timor-Leste showed 73% of soils were low or very low in P, as measured by Olsen P method with a critical limit of 15 ppm^14^.

Our protocol for plot soil sampling collection using bulk plot soil samples was found inappropriate, because at the three trial sites we found zero responses to soil fertilisation for soil variables despite the major agronomic reaction. Plot soil sampling was conducted on a bulk plot basis, whereas the application of fertilizer and biochar were confined to the transplant holes and not evenly distributed across the plots. Bulk plot soil sampling was not appropriate for unevenly distributed soil fertilisation.

The present study demonstrated fertilisation with rice husk biochar gave major yield responses across three sites and improved soil extractable concentrations of P, K and Zn at the acid soil site as seen in the soil incubation trial. This was unexpected considering that previously lignin-based biochars have shown no impact on soil P concentrations during soil incubation in the case of Li et al.^27^ and Novak et al.^28^. By contrast, poultry-based biochars have been shown to increase soil concentrations of P and K in other studies^28,29^. Regarding Zn, biochar addition to soils has been recorded to decrease extractable Zn in one agricultural trial^30^ and is known to reduce the toxic concentrations of Zn from contaminated soils. The rate of increase in the extractable soil concentrations of K, P and Zn, suggest that even if the soil had an extractable concentration of half the critical concentration, biochar rates of 5.1, 37 and 6 t ha^−1^ would be sufficient to increase the soil concentration above the critical values. Rates of application of 5–6 t ha^−1^ could be feasible in some horticulture applications in Timor-Leste, and therefore could be a practical way to increase soil fertility and reduce soil infertility.

The low pyrolysis temperature used to produce the biochar for this study is likely to be close to optimal for impacting soil properties. A review by Tomczyk et al.^31^ suggested “lower-temperature biochars, tend to have a better impact on soil fertility”. Biochar properties are dependent of the source material and the method of pyrolysis^10^. Rice husks at low-temperature pyrolysis were found by Prakongkep et al.^19^ to produce a mixture of amorphous and crystalline forms of silica resembling cristobalite and tridymite. They suggest “it provides a readily soluble form of both lime and plant nutrient elements so its use on acid, infertile soil should be encouraged”. It may also be possible to improve the fertilisation effect for necessary nutrients for specific soils with different source material, methods, speed and temperature of pyrolysis tailored for specific soil deficiencies. Ding et al.^22^ noted “the available P in the biochars produced at lower temperature was much higher than the high-temperature biochars”. The impact of pyrolysis and feedstock on Zn availability is largely unknown. Singh^32^ reported that in rice among plant parts the husk had the highest concentration of Zn, whereas the grain had the lowest. This suggests that, despite the overall soil Zn deficiency, the rice plant husk concentrates Zn preferentially in the husk.

Looking ahead, research is needed on economic rates of rice husk biochar fertilisation required and the longevity of its effects in Timor-Leste. In Ghana, SOC stocks doubled with rice husk biochar fertilisation but dropped thereafter, such that after 2 years biochar plots had only 31–38% higher SOC stocks compared to the control^33^. Widespread future use of rice husk biochar in soil fertilisation may be anticipated to increase carbon storage in Timor-Leste^34^. Amorphous (noncrystalline) silica is known as a major constituent of both rice husk and its biochar primarily as the water-insoluble cristobalite (SiO_2_)^19^. Given that rice hull biochar silica plays a role in ameliorating the deficiency of such nutrients such as Fe, Mn and Cu and plant disease resistance—as recently reviewed^23^, follow-up research on nutrient deficiency in Timor-Leste should embrace this mineral also.

In conclusion, building on literature from the tropics and a single year/site study in lowland Timor-Leste, this study was designed to test if biochar fertilisation would increase horticultural productivity in a range of tropical upland nutrient-poor soils differing in pH and also decrease acidity. We found marked increases in vegetable yields in response to fertilisation with rice husk biochar in three low-nutrient soils in Timor-Leste contrasting in soil pH. At the most acid site the yield response to biochar was among the highest recorded globally. In the soil incubation trial, we identified that the application of rice husk biochar alleviated soil deficiencies in P and Zn and gave a liming effect. The research has clarified that crop production in Timor-Leste is severely constrained by deficiencies in P and Zn and that the application of rice husk biochar is a promising avenue to fertilise the soil with these elements and increase crop productivity. As a result of the research farmers in Timor-Leste are now starting to apply rice husk biochar in their vegetable production.

## Methods

### Field study locations

Soil fertilisation field experiments were conducted at three locations in Timor-Leste—Caibada, Natarbora and Triloka—with contrasting soil types (Table 5). In summary, and with the soil analysis methodology given below, soil pH at the sites varied greatly from acidic (pH 5.2) at Triloka, through neutral at Natarbora (pH 7.3), to alkaline (pH 7.9) at Caibada. The acid Triloka site had the highest extractable Al content among the sites, was low in extractable Ca, Fe, Na, S and Zn, and had the greatest clay content among sites. In contrast, Caibada—the most alkaline and sandy site—had the highest %N and %C content and the greatest extractable Ca, Mg, Na and S concentrations. It also has the lowest extractable Al, Cu, Fe, and P. Natarbora soil with a nearly neutral pH had the lowest %N and %C values, but greatest Cu, Fe, K, P and Zn concentrations among sites.

**Table 5:** Geographic location and soil types including soil classification of three experimental field sites (Caibada, Natarbora and Triloka) in Timor-Leste with chemical analysis of surface soil (0–25 mm; control plots) of sites at harvest.

Variable	Caibada	Natarbora	Triloka
Latitude (°S)	8.4446	8.9898	8.5104
Longitude (°E)	126.4429	126.0655	126.3595
*Portuguese unit	VRPX	Acm	AR
*USDA classification	Humitropent	Usifluvent	Humitropent
*FAO classification	Vertisol/Gleysol	Calcaric Fluvisol	Ferrasol
Parent material	Coralliferous limestone	Recent alluvial deposits over limestone	Coralliferous limestone
Sand (%)	43.8 (1.69)	31.6 (2.19)	27.8 (2.26)
Silt (%)	40.4 (0.99)	53.0 (1.73)	43.4 (1.04)
Clay (%)	15.8 (0.70)	15.3 (0.45)	28.8 (1.26)
*pH	7.91	7.32	5.22
CEC	23.0	22.4	27.4
%N	0.262	0.174	0.24
%C	10.13	1.75	2.99
Al mg kg^−1^	< 3	444	1162
Ca mg kg^−1^	32,016	3974	3606
Cu mg kg^−1^	1.74	8.48	3.70
Fe mg kg^−1^	71	182	79
K mg kg^−1^	188	254	50
Mg mg kg^−1^	1006	247	465
Na mg kg^−1^	118	39	25
P mg kg^−1^	2.6	26.7	16.2
S mg kg^−1^	194.6	32.5	20.7
Zn mg kg^−1^	1.5	1.6	0.6

Values are means of three replicates of the control plots. Standard error values are in parentheses. *Portuguese soil map units from Garcia and Cardoso^35^; *USDA classification^36^; and *FAO classification^37^; * 1:5 soil to water suspension *; Al values at Caibada were below the detection limit.

### Experimental design of the field study

A soil fertilisation experiment was conducted at three sites: Caibada, Natarbora and Triloka. The five soil fertilisation treatments applied at each site were: 1. Control (no fertiliser application); 2. Local fertiliser regime (see details below); 3. Fertilisation with rice husk biochar (20 t ha^−1^ at Triloka and 30 t ha^−1^ at Caibada and Natarbora); 4. Addition of SP36 (28.8 kg P ha^−1^ and 4 kg S ha^−1^); and 5. The combination of biochar (Treatment 3) and SP36 (Treatment 4). The local fertiliser treatment varied by site according to local usage: At Caibada 10 t cow manure ha^−1^ applied; at Natarbora 100 kg NPK (15:15:15) with 2 t cow manure ha^−1^; and at Triloka 1.2 t cow manure ha^−1^. The rates of biochar were based on responses we had observed for other soils (unpublished data). Regarding P, the MAF recommended rate is 50 kg SP36 ha^−1^ and we used 28.8 kg P ha^−1^ and 4 kg S ha^−1^ (80 kg SP36 ha^−1^) to ensure a response to P. The experimental design varied with location. At Caibada and Triloka, the experiment included four horticultural crop species by five soil fertilisation treatments arranged in a split-plot design with crop species as main plots and the soil treatments as sub-plots. At Natarbora, the experiment included one horticultural crop species by five soil treatments in a randomised complete block design. Three replications were used throughout.

The horticulture crops for each site were selected based on their importance locally (Table 6). The four crops planted at Caibada were chili pepper and sweet pepper (both *Capsicum annuum* L.), Phaseolus bean *(Phaseolus vulgaris* L.*)* and carrot (*Daucus carota* L.). At Natarbora tomato (*Lycopersicum esculentum* Mill.) was planted. While at Triloka, tomato, soybean (*Glycine max* (L.) Mer.), chili pepper and yard-long bean (*Vigna unguiculata* subsp. *sesquipedalis* (L.) Verdc.) were grown. Locally available seed was sourced for each site. Vegetables were grown as irrigated dry-season crops. The previous wet-season crop was paddy rice at Caibada, fallow at Natarbora and upland mixed cropping at Triloka. Land preparation consisted of slashing and removal of weeds. Cultivation was by hand with the beds formed prior to planting. Experimental plots were 3 × 3 m beds raised approximately 10 cm high. Biochar and/or fertiliser was applied in the planting hole of transplanted crops or otherwise next to the seed. To plant seedlings, a planting hole approximately 25 cm diameter and 10 cm deep was dug, and a measured dose of fertiliser treatment was applied into the hole and mixed with the soil in the hole. A smaller hole was made to sow seeds. In the case of species with low planting density (3.3 plants m^−2^; Table 6), an application rate of 20/30 t ha^−1^, with the appropriate rate of rice husk biochar was applied to each planting hole. Seedlings were raised in nurseries and transplanted out after 2 to 3 weeks. Beans were direct sown. Irrigation was applied by hand to individual plants as required. One month after transplanting, plants were watered daily. No pest control was undertaken, and weeds were removed manually.

**Table 6:** Sowing dates (nursery or direct seeding) of the crops at the three sites.

Location	Crop	Sowing date	Plant density (plants m^−2^)
Caibada	Chili pepper	30-08-2018*	3.3
	Sweet pepper	24-08-2018*	3.3
	Phaseolus bean	25-07-2018	7.0
	Carrot	13-08-2018*	3.3
Natarbora	Tomato	18-08-2018*	3.3
Triloka	Chili pepper	02-06-2018*	3.3
	Tomato	03-06-2018*	3.3
	Yard-long bean	19-05-2018	7.0
	Soybean	19-05-2018	7.0

* Nursery sowing.

### Field growth and yield measurements

Plant height and leaf number on the main stem were recorded at 2 and 4 weeks after planting/ transplanting on three random plants per plot. Yield was measured by harvesting all the fresh matter production from plots except for soybean at Triloka where sun-dried yield was estimated. Yield components were calculated based on total yield and average fruit/seed size. Fruit/seed size was measured from three sample plants in each plot. In most crops there were multiple harvest with yield as the sum of the harvests.

### Soil sampling and analysis

Soil samples were collected from 5 locations to a depth of 250 mm in each plot in all locations after the harvest of the last crop. The five samples from each plot were then bulked, producing one soil sample per plot. Samples were air dried and thoroughly mixed at the soil laboratory in Dili. Approximately 200 g samples were sent from Dili to the laboratory in The University of Western Australia (UWA). Soil samples were analysed for pH and Olsen P in the MAF soil laboratory in Dili. Soil pH was measured in a 1:5 solution using a specific probe. Additional soil analyses were conducted at UWA after steam autoclaving at 121 °C for 30 min. Following autoclaving and grinding, soils were stored in a cool room at ambient temperature for 1 month prior to analysis. Concentrations of Al, Ca, Cu, Fe, K, Mg, Na, P, S and Zn were determined using Mehlich 3 extractions with inductively coupled plasma optical emission spectroscopy (ICP-OES) and total carbon and nitrogen were measured by dry combustion in a combustion analyser (Elementar, Langenselbold, Germany)^38^. Particle size analysis of the soils collected from the control plot from each site was determined by laser diffraction using a Mastersizer (Malvern, UK)^39^.

### Biochar production from rice husks

Rice husk biochar was made from air-dry husks sourced from a local rice mill. Rice husks were pyrolyzed with a modified Japan open retort^10^ consisting of a metal fly-screen rolled into a cylinder with diameter of 0.3 m and a length of 1.2 m. This chimney was placed on bare ground and a small fire using dry palm leaves was started at its base; and approximately 150 kg of rice husks then piled around. Pyrolysis took approximately four hours to complete. The temperature of pyrolysis was logged at 1 s time intervals with a long thermocouple (400 mm length) attached to a data logger. This was repeated on three batches with a maximum pyrolysis temperature of 413–450 °C. Four samples of rice husk biochar were sent to UWA for elemental analysis by wet digestion followed by Inductively Coupled Plasma Optical Emission Spectroscopy (ICP-OES), and for Mehlich 3 extraction (as above), and the results given in Table 7.

**Table 7:** Elemental analysis and Mehlich 3 extract analysis with means and standard errors for %N, %C, CEC and Mehlich 3 extractions for Al, Ca, Cu, Fe, K, Mg, Na, P, S and Zn of rice husk biochar (four samples with two replicates).

Variable	Elemental analysis	Extract analysis
N %	0.269	
Total C %	27.9	
CEC meq 100 g^−1^	18.7	
pH	8.9 (0.31)	
Al mg kg^−1^	1351 (589)	70 (11)
Ca mg kg^−1^	2972 (75)	1305 (274)
Cu mg kg^−1^	6.0 (0.3)	0.3 (0.05)
Fe mg kg^−1^	3960 (720)	116.5 (18)
K mg kg^−1^	6123 (113)	4144 (186)
Mg mg kg^−1^	1460 (235)	435.7 (48)
Na mg kg^−1^	397 (49)	230 (27)
P mg kg^−1^	1525 (100)	561.3 (48)
S mg kg^−1^	569 (71)	93.5 (35)
Zn mg kg^−1^	43 (2.9)	7.3 (0.3)

Standard error values are in parentheses.

### Soil incubation pot study

A soil incubation pot study without plants was conducted to investigate how fertilisation with rice husk biochar affects soil pH and available P. The study used soil collected within 5 m of the acid field site Triloka (0–250 mm depth), where the response to biochar application was greatest, and was conducted at the Ministry of Agriculture and Fisheries compound at Comoro, Dili. The experimental design was completely randomized with four rates of rice husk biochar (0, 5, 10 and 20 t ha^−1^) with five replications. Free-draining pots (200 mm diameter by 200 mm height) were filled with 5 kg soil in late December 2018. Appropriate rates of biochar were added to the soil throughout the full depth of the pot. Pots were placed under ambient temperature and rainfall for 26 weeks with drainage through the rainy season and start of the dry season (January to July 2019) in Dili without seeding, for which period a total of 499.5 mm rainfall was received (Supplementary Table 1). Pots were regularly weeded throughout.

Over the 6-month study, soil samples were collected from each pot to a depth of 100 mm every second week for analysis, providing 13 sample dates (including the initial soil sample). Soil samples were analysed for soil pH as described above. Soil samples collected at the final two sample dates were analysed at UWA for %C, %N, EC, Al, Ca, Cu, Fe, K, Mg, Na, P, S, and Zn as described above. Each laboratory used the same soil samples for analysis.

### Data analysis

Analyses of variance and correlations were conducted with GenStat Discovery version 19 (VSN International). The GENSTAT repeated measures procedure AREPMEASURES was used to conduct analyses of variance for soil pH and results of Mehlich 3 extracts from the soil incubation study.

### Legislative compliance

 All plant experiments were conducted in accordance to relevant institutional, national, and international guidelines and legislation.

## Supplementary Information

Supplementary Tables.

## Data Availability

All data generated or analyzed during this study are included in this published article.
