# Supplementary material for: Productivity gains in vegetables from rice husk biochar application in nutrient-poor soils in Timor-Leste
Source: Sci Rep. 2023 Jul 5;13:10858. doi: 10.1038/s41598-023-38072-2 (PMC10322997; doi:10.1038/s41598-023-38072-2)
Supplement: Supplementary file 1 — Supplementary Tables. [file 41598_2023_38072_MOESM1_ESM.docx]

**Supplementary Information**

**Title:** Productivity gains in vegetables from rice husk biochar application in nutrient-poor soils in Timor-Leste

**Journal:** Journal of Soil Science and Plant Nutrition

**Author names:** Rob Williams, Joao Bosco Belo, Julieta Lidia, Salvador Soares, Decio Ribeiro, Celestino L. Moreira, Luis Almeida, Louise Barton and William Erskine*

**Affiliations and email of corresponding author:** WE - Institute of Agriculture/ School of Agriculture and Environment, University of Western Australia, 35 Stirling Hwy, Nedlands 6009 WA Australia. ***** william.erskine@uwa.edu.au

**Supplementary Table S1** Monthly rainfall totals (mm) in Dili 2019 during the soil incubation experiment

| Month | Rainfall (mm) |
| --- | --- |
| January | 165.3 |
| February | 91.6 |
| March | 194.0 |
| April | 8.8 |
| May | 30.4 |
| June | 0.6 |
| July | 8.8 |

| **Supplementary Table S2** Means of soil pH and Mehlich 3 soil extracts for Al, Cu, Fe, K, Mg, Na, P, S and Zn from bulk plot soil samples after harvest of fertiliser treatments (Treat: control, local fertiliser, biochar, SP36, and biochar + SP36 in combination) means at three sites (Caibada, Natarbora and Triloka) in Timor-Leste in 2018. Al values at Caibada were below the detection limit | | | | | | | | | | | |
| --- | --- | --- | --- | --- | --- | --- | --- | --- | --- | --- | --- |
| Site/Treatment | Soil pH | Al mg kg^-1^ | Cu mg kg^-1^ | Ca mg kg^-1^ | Fe mg kg^-1^ | K mg kg^-1^ | Mg mg kg^-1^ | Na mg kg^-1^ | P mg kg^-1^ | S mg kg^-1^ | Zn mg kg^-1^ |
| Caibada |  |  |  |  |  |  |  |  |  |  |  |
| Control | 7.91 | <3 | 1.74 | 32016 | 71 a | 188 | 1006 | 118 | 2.6 a | 194.6 | 1.5 |
| Local fertiliser | 8.12 | <3 | 2.19 | 31538 | 71 a | 191 | 989 | 111 | 3.4 a | 184.3 | 2.1 |
| Biochar | 7.99 | <3 | 1.8 | 32007 | 66 a | 219 | 947 | 99 | 2.2 a | 170.1 | 1.7 |
| SP36 | 8.05 | <3 | 2.06 | 31813 | 71 a | 195 | 998 | 115 | 2.7 a | 191.2 | 1.5 |
| Biochar + SP36 | 8.07 | <3 | 1.98 | 31765 | 59 a | 196 | 926 | 111 | 2.7 a | 163.6 | 1.6 |
| Site mean | 8.03 c | <3 | 1.95 a | 31828 c | 68 | 198 b | 973 c | 111 b | 2.7 | 181 b | 1.7 b |
| Natabora |  |  |  |  |  |  |  |  |  |  |  |
| Control | 7.32 | 444 | 8.48 | 3974 | 182 bc | 254 | 247 | 39 | 26.7 a | 32.5 | 1.6 |
| Local fertiliser | 7.58 | 416 | 8.26 | 4453 | 171 bc | 248 | 217 | 30 | 26.2 a | 27.5 | 1.5 |
| Biochar | 7.27 | 405 | 7.65 | 3782 | 147 b | 261 | 233 | 27 | 32.7 a | 29.7 | 3.5 |
| SP36 | 7.37 | 424 | 7.98 | 3994 | 166 bc | 274 | 247 | 28 | 46.2 ab | 30.5 | 2.1 |
| Biochar + SP36 | 7.19 | 457 | 8.15 | 4102 | 221 c | 264 | 302 | 42 | 187.7 b | 30.3 | 2.4 |
| Site mean | 7.35 b | 429 a | 8.10 c | 4061 b | 177 | 260 c | 249 a | 33 a | 63.9 | 30.1 a | 2.2 b |
| Triloka |  |  |  |  |  |  |  |  |  |  |  |
| Control | 5.22 | 1162 | 3.7 | 3606 | 79 a | 50 | 465 | 25 | 16.2 a | 20.7 | 0.6 |
| Local fertiliser | 5.1 | 1161 | 3.29 | 3370 | 71 a | 48 | 452 | 25 | 16.2 a | 19.1 | 0.5 |
| Biochar | 4.95 | 1210 | 3.57 | 3570 | 83 a | 66 | 484 | 27 | 15.2 a | 20.3 | 0.6 |
| SP36 | 5.1 | 1190 | 3.53 | 3588 | 77 a | 50 | 441 | 24 | 15.0 a | 23.0 | 0.5 |
| Biochar + SP36 | 5.18 | 1188 | 3.69 | 3706 | 85 a | 56 | 475 | 27 | 14.4 a | 20.6 | 0.6 |
| Site mean | 5.11 a | 1182 b | 3.56 b | 3568 a | 79 | 54 a | 463 b | 26 a | 15.4 | 20.7 a | 0.6 a |
| Prob F Treat | 0.123 | 0.492 | 0.747 | 0.85 | 0.523 | 0.193 | 0.965 | 0.867 | 0.329 | 0.84 | 0.418 |
| Prob F Site | <0.001 | <0.001 | <0.001 | <0.001 | <0.001 | <0.001 | <0.001 | <0.001 | <0.001 | <0.001 | <0.001 |
| Prob F Site x Treat | 0.101 | 0.221 | 0.321 | 0.508 | 0.035 | 0.949 | 0.566 | 0.986 | 0.024 | 0.985 | 0.082 |
| CV % | 2.61 | 5.9 | 12.5 | 3.23 | 23.1 | 17.2 | 13 | 40.1 | 243 | 42.8 | 51.1 |

Treatments followed by the same letter and in the same column are not significantly different from each other (P<0.05). For Natabora samples are from 3 replicates of only tomato plots (n=3). For Caibada and Triloka, soils samples are from 3 replicates of two species (n=6)
